# Evaluation of chromosomal abnormalities in the postnatal cohort: A single‐center study on 14,242 patients

**DOI:** 10.1002/jcla.24997

**Published:** 2023-12-19

**Authors:** Hilal Akalin, Izem Olcay Sahin, Seyma Aktas Paskal, Busra Tan, Ezgi Yalcinkaya, Mikail Demir, Mustafa Yakubi, Busra Ozguc Caliskan, Ozlem Gokce Ekinci, Mehmet Ercan, Tugce Yasar Kucuk, Gizem Gokgoz, Aslihan Kiraz, Huseyin Per, Mahmut Tuncay Ozgun, Numan Baydilli, Yusuf Ozkul, Munis Dundar

**Affiliations:** ^1^ Department of Medical Genetics, Faculty of Medicine Erciyes University Kayseri Türkiye; ^2^ Department of Pediatric Neurology, Faculty of Medicine, Children's Hospital Erciyes University Kayseri Türkiye; ^3^ Department of Obstetrics and Gynecology, Faculty of Medicine Erciyes University Kayseri Türkiye; ^4^ Department of Urology, Faculty of Medicine Erciyes University Kayseri Türkiye

**Keywords:** chromosomal abnormality, gender, karyotype, postnatal

## Abstract

**Background and Aim:**

Chromosomal analysis is a laboratory technique used to examine the chromosomes of an individual, offering insights into chromosome numbers, structures, and arrangements to diagnose and comprehend genetic diseases. This retrospective study provides a comprehensive understanding of the distribution by indications in a large cohort of 14,242 patients and the frequency of chromosomal abnormalities in different clinical populations.

**Method:**

The study examined various indications for karyotype evaluation, with recurrent pregnancy loss being the most common indication, followed by intellectual disability, dysmorphic features, congenital anomalies, and developmental delay.

**Results:**

The overall chromosomal abnormality rate was found to be 5.4%, with numerical abnormalities accounting for the majority of cases (61.7%). Trisomies, particularly trisomy 21, were the most frequent numerical abnormalities. In terms of structural abnormalities, inversions and translocations were the most commonly identified. The rates of chromosomal anomalies varied in specific indications such as amenorrhea, disorders of sex development, and Turner syndrome. The study also highlighted significant differences between males and females in the presence of chromosomal abnormalities across certain indications. Males exhibited a higher incidence of chromosomal abnormalities in cases of Down syndrome and infertility, whereas females showed higher abnormalities in terms of recurrent pregnancy loss.

**Conclusion:**

While this study provides valuable insights into the frequency and distribution of chromosomal abnormalities, it has limitations, including its retrospective design and reliance on data from a single medical genetics department. Nevertheless, the findings emphasize the importance of karyotype analysis in diagnosing chromosomal disorders and providing appropriate management, while also pointing to potential gender‐related variations in chromosomal abnormalities that warrant further investigation.

## Graphical abstract

This retrospective study examined various indications for classical karyotype evaluation in a large cohort of 14,242 patients. The overall chromosomal abnormality rate was 5.4%. The most common chromosomal abnormalities were Down syndrome (trisomy 21), inversions, and translocations. Males were more likely to have chromosomal abnormalities in cases of Down syndrome and infertility, while females were more likely to have chromosomal abnormalities in cases of recurrent pregnancy loss.

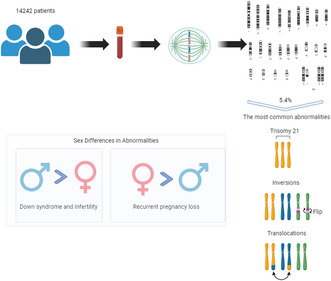

## 1 INTRODUCTION

Chromosomal abnormalities have a great burden as a cause of early pregnancy loss, fetal malformations, and stillbirth. Apart from these, chromosomal abnormalities increase this burden as they often cause intellectual disability, malformations, and infertility. The incidence of all chromosomal abnormalities in live‐born infants has been reported as 1 in 153 births in the data obtained by combining data from a series of studies with newborns.^1^ The prevalence of syndromic chromosomal abnormalities including all cases (fetal deaths/stillbirths from 20 weeks gestation, live births, and termination of pregnancy for a congenital anomaly) was reported as 45.28 per 10,000 births in 2019, according to EUROCAT.^2^ Karyotyping is one of the most preferred methods for detecting structural and numerical abnormalities. Deletions, duplications, balanced or unbalanced translocations, insertions, and inversions are examples of structural abnormalities.^3^ These anomalies may include autosomes or sex chromosomes.^4^

The higher incidence of chromosomal abnormalities demonstrates the importance of cytogenetic evaluation in patients with dysmorphic features and congenital anomalies.

Karyotype analysis remains a useful tool for the diagnosis of major chromosomal abnormalities. However, since array technology is a more sensitive and high‐resolution method, it provides a great advantage in detecting small‐scale genetic changes and performing a more detailed genetic analysis.^5^ While array technology offers higher resolution, cytogenetic investigation remains the primary method for detecting mosaicism.^6^ Therefore, using both methods together in clinical practice enables genetic diagnosis and counseling processes to be carried out more effectively and comprehensively. The findings suggest that chromosomal analysis should be used in the search for patients with genetic disorders of unknown origin to confirm clinical diagnosis and appropriate medical care after genetic counseling and management.^7^ Chromosomal abnormalities affect at least 7.5% of all pregnancies. Most of these abnormalities resolve spontaneously, and the frequency is 0.6% in live births.^8^ The high rate of chromosomal abnormalities (32.2%) found in a study conducted on a population of 4216 people indicates the importance of cytogenetic evaluation in patients with clinical abnormalities.^9^ Today, structural and numerical anomalies in one or more chromosomes are among the major causes of intellectual disability, and approximately half of the chromosomal causes of intellectual disability are numerical chromosome anomalies.^10^

Because most chromosomal abnormalities are incompatible with life, the incidence of chromosomal abnormalities is lower in live births than in miscarriages, second‐trimester fetuses, or stillbirths. The clinical consequences of some chromosomal anomalies may occur at later ages. Patients with chromosomal abnormalities that cause symptoms such as amenorrhea, recurrent pregnancy loss, and infertility can be diagnosed in the pubertal and post‐pubertal period, which is usually the period when their complaints occur.^11^, ^12^, ^13^ Many studies in the literature have revealed the incidence of prenatal chromosomal anomalies.^14^, ^15^, ^16^, ^17^, ^18^, ^19^

However, few studies have focused on postnatal chromosomal abnormalities.^20^, ^21^ In this study to fill this gap in the literature, we aimed to reveal the postnatal chromosomal anomaly rate in our cohort. For this purpose, chromosome analysis results obtained from peripheral blood samples of 14,242 patients who applied to Erciyes University Medical Genetics Department were examined. Chromosome analysis results were categorized and evaluated. As a result of the evaluation, the prevalence and types of chromosomal abnormalities among the patients were determined. The results obtained were compiled to contribute to the genotype–phenotype correlation.

## 2 METHODS

In this retrospective study, the chromosome analysis results of 14,242 patients who applied to Erciyes University Medical Genetics Department between 2013 and 2020 were evaluated. Our cohort consists of infants who need confirmation of their prenatal diagnostic test results, infants suspected of a chromosomal abnormality that did not receive proper antenatal care, infants who had an antenatal suspicion of chromosomal abnormality that did not undergo prenatal karyotyping for various reasons, pediatric and adult patients with a suspected chromosomal abnormality that presented postnatally. We performed descriptive statistics to summarize postnatal cohort data and describe the characteristics of the study population, including the frequency and distribution of chromosomal abnormalities. In addition, the Pearson chi‐square test was used to determine whether the chromosomal aberrations associated with the indications differed between males and females. This study included patients presenting with various indications, such as amenorrhea (60 cases), abnormal sexual differentiation (79 cases), Turner syndrome (307 cases), congenital anomaly/dysmorphic feature/growth retardation/intellectual disability (4120 cases), Down syndrome (467), trisomy 13 (seven cases), trisomy 18 (22 cases), infertility (2585 cases), recurrent miscarriage (6021 cases), Klinefelter syndrome (62 cases), and miscellaneous (512 cases). The age of the patients ranged from newborn to 65 years with a mean of 22.08 years, 1590 (11.1%) cases were below 1 year age. Of the 14,242 patients, 6795 (47.7%) were females and 7447 (52.3%) were males. The routine cytogenetic analysis procedure was applied to the samples for cytogenetic analysis.^22^, ^23^

This study was approved by the Erciyes University Clinical Research Ethics Committee (Approval number: 2021/308).

## 3 RESULTS

When 14,242 patients evaluated in the study were classified according to their indications, it was observed that the majority of the cases were comprised of the recurrent pregnancy loss group, with a rate of 42.3%. The second group was the intellectual disability/dysmorphic features/congenital anomalies/developmental delay and similar groups at a rate of 28.9%, and the third group was the infertility group at a rate of 18.2%. These three groups constitute 89.4% of the total cases. Table 1 depicts the distribution of cases based on clinical indications.

**TABLE 1:** Distribution of cases by clinical indications for referral for cytogenetic analysis.

	The frequency of cases referred (*n*)	Percent (%)	Frequency of cases with chromosomal abnormalities (*n*)	Percent (%)
Primary amenorrhea	60	0.4	8 (13.3%)	1.02
Disorders of sex development	79	0.6	15 (18.9%)	1.93
Turner syndrome	307	2.2	53 (17.2%)	6.82
Intellectual disability/dysmorphic features/congenital anomalies/developmental delay and so forth	4120	28.9	58 (1.4%)	7.59
Down's syndrome	467	3.3	275 (58.8%)	35.39
Trisomy 13	7	0.0	7 (100%)	0.9
Trisomy 18	22	0.2	22 (100%)	2.83
Klinefelter's syndrome	62	0.4	24 (38.7%)	3.08
Infertility	2585	18.2	138 (5.33%)	17.76
Repeated abortions	6021	42.3	163 (2.7%)	20.97
Miscellaneous	512	3.6	15 (2.7%)	1.67
Total	14,242	100.0	778 (5.46%)	100

When the results of the chromosomal analysis were evaluated, the chromosomal anomaly was detected in 778 patients, and the total chromosomal abnormality rate was found to be 5.4%. While 61.7% of this was numerical chromosomal anomalies, 38.3% were structural chromosomal anomalies. Of the 480 numerical abnormalities, 291 cases were trisomies, and 32 cases had X monosomies. Inversion and translocation were the most common structural chromosomal anomalies. The frequencies of the different forms of abnormal karyotypes are shown in Tables 2, 3, 4.

**TABLE 2:** Frequency of numerical chromosome abnormalities and structural chromosome rearrangements.

	Frequency (*n*)	Percent (%)
Numerical chromosome abnormalities	480	61.7
Structural chromosome rearrangements	298	38.3
Total	778	100

**TABLE 3:** Frequency of numerical chromosome abnormalities.

Sex chromosome	Frequency (*n*)	Autosomal chromosome	Frequency (*n*)
45,X	32	47,XX,+21	113
47,XYY	8	47,XY,+21	149
47,XXX	6	47,XY,+13	4
47,XXY	97	47,XX,+13	3
47,XXY/46,XY	1	47,XX,+18	14
45,X/47,XYY	1	47,XY,+18	8
45,X/46,XY	5		
45,X/46,XX	23		
47,XXX/46,XX	4		
45,X/46,XX/47,XXX	8		
45,X/46,XY/47,XXY	1		
48,XXXX	1		
49,XXXXX	1		
49,XXXXY	1		
Total	189		291

**TABLE 4:** Frequency of structural chromosomal rearrangements.

	Frequency (*n*)	Percent (%)
Isochromosome X	10	3.2
Deletions	28	9.4
Inversions	137	46
Ring	2	0.6
Marker	9	3
Translocation	96	32.2
46,XX‐Male	5	1.6
46,XY‐Female	12	4
Total	298	100

### 3.1 Amenorrhea – disorders of sex development – Turner syndrome groups

#### 3.1.1 Amenorrhea

When the results of 60 patients who applied with the indication of primary amenorrhea were evaluated, abnormal karyotype structure was detected in 13.3% of them (Table S1).

#### 3.1.2 Disorders of sex development

A total of 79 (0.6%) cases with disorders of sex development were referred for karyotyping. Numerical or structurally abnormal karyotype structure was observed in 15 (19.1%) of the patients (Table S2).

#### 3.1.3 Turner syndrome

Of the 307 cases referred for Turner syndrome, 53 (17.3%) were found to have chromosomal abnormality. Classical 45,X monosomy was the most frequent anomaly (30 cases), and the other abnormalities were either mosaics (15 cases) or structural X abnormalities, including long‐arm isochromosome (nine cases), long‐arm deletion (three cases), mosaics marker chromosome (one case) and 46 XY‐Female (one case). Of the 28 mosaics, eight had a Y chromosome component (46,XY/45,X). Chromosomal abnormalities in Turner syndrome are shown in Table S3.

#### 3.1.4 Intellectual disability/dysmorphic features/congenital anomalies/developmental delay, and so forth‐Edwards syndrome – Patau syndrome groups

Of the 4120 cases referred for intellectual disability, dysmorphic features, congenital anomalies, developmental delay, and so forth, 58 cases (1.40%) had chromosomal abnormalities. These abnormalities include 22 balanced translocations, 10 deletions, 10 inversions, six Klinefelter's syndromes, two rings, four markers, one 47,XXX, one 48,XXXX, one 49,XXXXX, and one 49,XXXXY.

Also, the results of seven patients evaluated with the indication trisomy 13 and 22 patients evaluated with the indication trisomy 18 were found to be 100% compatible with the indication.

#### 3.1.5 Down syndrome

Of the 467 patients evaluated with the indication of Down syndrome, 242 were female and 225 were male. The anomaly rate was 58.8% in the Down syndrome group. 89.2% of patients with Down syndrome were detected under 1 year of age. When evaluated according to gender, this rate was 50% and 68.4% for females and males, respectively. It was determined that 262 patients had classical Down Syndrome, 10 patients had translocation, two patients had marker chromosomes, and one patient had 47,XXX. In our study, the most common translocation was t(21;21). Chromosomal abnormalities in Down syndrome are shown in Table S4.

#### 3.1.6 Klinefelter syndrome

Sixty‐two patients were evaluated with the indication of Klinefelter syndrome, 47,XXY anomaly was found in 23 patients (37.1%), and 47,XYY anomaly was found in one patient (Table S5).

#### 3.1.7 Infertility

Chromosome analysis was performed on a total of 2585 patients, 1051 females and 1534 males, who applied with the indication of infertility. The chromosomal anomaly was detected in 138 patients. The total anomaly rate (including inv(9)) was found to be 5.33%. The chromosome analysis of the patients evaluated with an infertility indication revealed 19 patients with translocation (eight of whom had Robertsonian translocation), four patients with deletion, 25 patients with inversion (23 of whom had inv(9)), 89 patients with the numerical anomaly, and one patient with marker chromosome. Polymorphism was detected in 76 patients and the most common polymorphism was inv(9). The second most common was 16qh+. In Table S6, chromosomal abnormalities in infertility were shown.

#### 3.1.8 Recurrent pregnancy loss

Chromosome analysis was performed on a total of 6021 patients, 3154 female, and 2867 male, who applied with the indication of recurrent pregnancy loss. The chromosomal anomaly was detected in 163 patients. The total anomaly rate (including inversion 9 and inversion Y) was found to be 2.7%. A Robertsonian translocation was found in 17 patients, with the most common Robertsonian translocation being rob(13;14) (Table S7). Polymorphism was detected in 213 patients, and the most common polymorphism was inv(9). The second most common was 9qh+ (Table S8).

#### 3.1.9 Chromosomal abnormality according to gender

According to the chi‐square test results, the presence of chromosomal abnormalities significantly differs between males and females in Down syndrome, infertility, and recurrent pregnancy loss indications (Table 5). Males had more chromosomal abnormalities than females for Down syndrome and infertility, whereas females had more chromosomal abnormalities for syndromes and recurrent pregnancy loss. There was no difference between males and females in terms of classical trisomy 21 and translocation trisomy 21 for Down syndrome. For infertility and recurrent pregnancy loss, there was no difference between males and females in terms of gender and autosomal chromosome abnormality. Of the 242 patients presenting with a preliminary diagnosis of Down syndrome, 118 had classical trisomy 21 or translocation trisomy 21. Two patients had chromosomal abnormalities other than chromosome 21. The analysis was performed with 118 patients with confirmed Down syndrome and 121 patients without chromosomal abnormalities.

**TABLE 5:** Presentation of results from chi‐square analysis of chromosomal abnormality according to gender.

	Chromosomal abnormality		*χ* ^2^ (df)	*p*
	Yes	No		
	*N* (%)	*N* (%)		
Down syndrome			17.379 (1)	0.000
Female	118 (49.4%)	121 (50.6%)		
Male	154 (68.4%)	71 (31.6%)		
Infertility			37.204 (1)	0.000
Female	15 (1.5%)	996 (98.5%)		
Male	100 (6.7%)	1398 (93.3%)		
Recurrent pregnancy loss			17.944 (1)	0.000
Female	58 (1.9%)	2975 (98.1%)		
Male	18 (1.3%)	2759 (98.7%)		

Our current study was compared to similar research published in our country and worldwide in Table 6.

**TABLE 6:** Comparison of the current study with the others.

	Our study		9		24		23	
	Total referrals	Abnormal karyotype	Total referrals	Abnormal karyotype	Total referrals	Abnormal karyotype	Total referrals	Abnormal karyotype
Primary amenorrhea	60 (0.4%)	8 (13.3%)	342 (6.0%)	56 (16.4%)	31 (3.1%)	4 (12.9%)	‐	‐
Disorders of sex development	79 (0.6%)	15 (18.9%)	162 (2.9%)	22 (13.6%)	45 (4.5%)	2 (4.4%)	98 (11.4%)	5 (5.1%)
Turner syndrome	307 (2.2%)	53 (17.2%)	486 (8.5%)	95 (19.6%)	47 (4.7%)	5 (10.6%)	287 (33.4%)	51 (17.7%)
Intellectual disability/dysmorphic features/congenital anomalies/developmental delay and so forth	4120 (28.9%)	58 (1.4%)	568 (10.0%)	30 (5.3%)	314 (31.4%)	29 (46%)	117 (13.6%)	13 (11.1%)
Down's syndrome	467 (3.3%)	275 (58.8%)	1048 (18.4%)	557 (53.2%)	70 (3.5%)	60 (85.7%)	357 (41.6%)	302 (84.5%)
Trisomy 13	7 (0.0%)	7 (100%)						
Trisomy 18	22 (0.2%)	22 (100%)						
Klinefelter's syndrome	62 (0.4%)	24 (38.7%)	364 (6.4%)	92 (25.3%)	19 (1.9%)	6 (31.6%)	‐	‐
Infertility	2585 (18.2%)	138 (5.33%)	134 (2.4%)	21 (15.7%)	19 (1.9%)	6 (31.6%)	‐	‐
Repeated abortions	6021 (42.3%)	163 (2.7%)	1892 (33.3%)	44 (2.3%)	273 (27.3%)	10 (3.6%)	‐	‐
Miscellaneous	512 (3.6%)	15 (2.7%)	330 (5.8%)	0	‐	‐	‐	‐
Total	14,242 (100.0%)	778 (5.4%)	5688 (100.0%)	917 (16.1%)	1000 (100%)	135 (13.4%)	859 (100%)	371 (43.1%)

## 4 DISCUSSION

The results of the study provide a comprehensive understanding of the distribution of patients according to their indications and the frequency of chromosomal abnormalities within various clinical populations.

The indications for karyotype evaluation varied in this study, with recurrent pregnancy loss being the most common indication (42.3%), followed by intellectual disability, dysmorphic features, congenital anomalies, and developmental delay group (28.9%). These results are consistent with previous research that identified developmental disorders and recurrent pregnancy loss as the two primary indications for chromosomal analysis.^24^ The infertility group comprised 18.2% of the cases. These underscore the significance of genetic examinations in identifying the etiology of these indications and possible chromosomal causes, as well as in providing appropriate counseling.

The overall chromosomal abnormality rate was found to be 5.4% (748/14242), with numerical abnormalities accounting for 61.7% and structural abnormalities accounting for 38.3% of the cases. Polipalli et al.^23^ found 43.1% in 859 patients, Al Husain and Zaki^24^ found 13.4% in 1000 patients, and Pal et al.^21^ found 12.23% in 2215 patients. However, the detection of chromosomal anomalies in only 5.4% of the patients referred revealed the importance of clinicians making a good clinical evaluation to use the resources correctly before directing the patients to chromosomal analysis. Trisomies were the most frequent numerical abnormalities in this study, with trisomy 21 (Down syndrome) being the most common among them. The high proportion of numerical abnormalities, particularly trisomies, is consistent with the well‐established association between advanced maternal age and an increased risk of numerical chromosomal anomalies.^25^ Regarding the frequency of structural and numerical chromosomal abnormality, the results are consistent with a previous study.^26^ It is worth noting that the percentage of cases with numerical abnormalities in this study (61.7%) is lower than the reported rate of 80% for numerical abnormalities.^9^ The disparity may be explained by variations in patient groups and study sample sizes.

Inversions and translocations were the most frequently identified abnormalities concerning structural chromosomal anomalies. Common structural rearrangements in the human genome, such as inversions and translocations, have been identified, with varying clinical significance depending on the specific chromosomal regions involved and the presence of accompanying imbalances.^27^ This study's finding about structural chromosomal abnormalities emphasizes the importance of thorough karyotyping for individuals with clinical indications because even balanced rearrangements can have phenotypic consequences or affect reproductive outcomes. A genome‐wide study in patients with developmental delay and/or multiple congenital anomalies reported that the vast majority of apparently balanced de novo chromosomal rearrangements had cryptic instability, usually a deletion or duplication at breakpoint sites.^28^ Carriers of balanced translocations have an increased risk of recurrent abortion and/or having children with congenital abnormalities.^29^

The rates of chromosomal anomalies varied in certain indications, such as amenorrhea, disorders of sex development, and Turner syndrome. 13.3% of patients evaluated with primary amenorrhea had an abnormal karyotype. Previous studies have reported that 12%–42% of patients with primary amenorrhea have abnormal karyotypes, and this may be due to differences in the selection criteria of the patients.^13^ Primary amenorrhea can arise from endocrine disorders, structural and environmental factors, and genetics. When exploring its genetic etiology, cytogenetic abnormalities, should not be neglected as a primary investigation.^30^ In cases where chromosomal analysis yields normal results, advanced molecular research and genetic counseling become of significant importance.

In patients referred for disorders of sex development, the majority (80.9%) did not exhibit numerical or structural chromosomal anomalies. Comparing the findings of this study with literature related to this indication, it is observed that the prevalence of chromosomal abnormalities in disorders of sex development (18.5%) aligns with a previous report.^31^ This finding is consistent with previous reports indicating that many cases of disorders of sex development are not associated with detectable chromosomal abnormalities.^32^ It is important to consider, though, that certain cases can entail submicroscopic rearrangements or mutations in the genes responsible for sex determination and differentiation, which might not be detectable by conventional karyotyping. Therefore, in this indication, advanced genetic tests such as microarray and next‐generation sequencing should be evaluated depending on the patient.

17.2% of patients who were referred for Turner syndrome exhibited chromosomal abnormalities; the most common abnormality in this study was classic 45,X monosomy. This finding is consistent with other research showing that monosomy X causes the majority of cases of Turner syndrome.^9^, ^33^ The finding of mosaicism and structural X abnormalities highlights the significance of the thorough chromosomal study in Turner syndrome cases even more because these variations can change the clinical presentation as well as the management of those who are affected. Our findings are compatible with previous studies that highlighted the importance of using molecular genetic techniques to find submicroscopic chromosomal abnormality in Turner syndrome cases.^34^

In patients presenting with intellectual disability, dysmorphic features, congenital anomalies, and developmental delay, the overall rate of chromosomal abnormalities was 1.4%. In this patient population, chromosomal microarray and next‐generation sequencing are recommended for patients with no anomalies detected. Among the anomalies found were balanced translocations, deletions, inversions, and sex chromosomal aneuploidies. However, karyotype analysis alone has been reported to explain causality in 3%–5% of children with moderate to severe intellectual disability, excluding Down syndrome.^35^ This is even though the discovery of these chromosomal abnormalities in people with developmental disorders supports the value of karyotyping in establishing a genetic diagnosis and providing accurate genetic counseling. Therefore, in this indication, advanced genetic tests such as microarray and next‐generation sequencing should be evaluated depending on the patient.

Further validating the accuracy and reliability of the chromosomal analysis performed in this study were the findings of certain chromosomal abnormalities, such as trisomy 13, trisomy 18, and Down syndrome. Trisomy 13 and trisomy 18 are well‐recognized chromosomal abnormalities associated with pathognomonic dysmorphic features and a high rate of prenatal diagnosis.^36^ The efficacy of the cytogenetic analysis method used in this study is demonstrated by the results' ideal compliance with the indications in these cases. Genetic counseling, including the risk of recurrence, is of great importance for families with children with these two important syndromes, where long‐term survival is not expected.

Chromosomal anomalies were observed in 58.8% of the Down syndrome patients, with 89.2% of these patients being identified before the age of 1 year. While classical trisomy 21 is the most common form of Down syndrome (95.3%), translocations involving chromosome 21 have also been identified in some cases. Previous studies consistent with this result reported 53.2%^9^ and 49.1%,^8^ while higher frequencies of 85.7%,^24^ 72.8%,^33^ and 84.5%^23^ have also been reported in Down syndrome. These high rates may be due to the low preferences of families for prenatal screening, or legal and religious decisions that affect the outcome if prenatal screening results confirm the disease. Our study aligned with another study that reports that the frequency of translocation is 3.1% and the frequency of classical trisomy 21 is 96%.^33^ The finding of a variety of chromosomal abnormalities in Down syndrome patients emphasizes the importance of a comprehensive cytogenetic investigation for accurate diagnosis, medical management, and genetic counseling, including the risk of recurrence and family segregation. The analysis of patients with Klinefelter syndrome revealed a 38.7% abnormality rate with the presence of 47,XXY and 47,XYY anomalies. These results support the extensively reported chromosomal abnormalities associated with Klinefelter syndrome.^24^, ^37^ Patients with 47,XYY may be confused with Klinefelter syndrome in clinical evaluation due to tall stature, behavioral problems, and abnormal spermogram.^38^ Therefore, it is essential to make cytogenetic distinctions for these patients. The identification of these anomalies highlights the significance of chromosomal analysis for individuals suspected of having Klinefelter syndrome since it allows a precise diagnosis alongside appropriate management.

The rate of chromosomal anomalies in patients with infertility was 5.33%, and various types of abnormalities were reported. The abnormalities identified comprised translocations, deletions, inversions, numerical anomalies, and marker chromosomes. In the literature, 5.1%^39^ and 5.5%^40^ rates align with this result. Infertile males in our cohort had an autosomal translocation rate of 1.06%, which was consistent with one study's finding (1.07%), while infertile females had a rate of 0.3%, which was lower than this same study (1.28%).^41^ The most common anomaly in female patients with chromosomal anomalies was 45,X/46,XX. While the numerical X mosaism rate was 0.9% in our study, the literature reported the prevalence of numerical X mosaicism in infertile females as 0.3%^41^ and 2.8%.^42^ This difference is may due to the cut‐off value used to determine the real mosaicism.^41^ In our study, male patients most frequently have the abnormality 47,XXY which results in Klinefelter syndrome, the most prevalent genetic cause of male infertility.^43^ This rate varies between 3% and 4%, according to the literature.^44^ In infertile individuals who do not have Klinefelter syndrome or 47,XYY karyotype, it should be considered that microdeletions in the AZF region of the Y chromosome may also be the cause of infertility, apart from aneuploidy.^45^ Apart from these, polymorphism was detected in 76 of the infertile patients. The most common polymorphism among these polymorphisms was inv(9). Today, discussions about inv(9) polymorphism still continue, and clinical studies have shown that the frequency of this variant is increased compared to the normal population.^46^ In our study, consistent with the literature,^47^ inv(9) was detected more frequently in women than in men. Because chromosomal abnormalities might affect reproductive outcomes, these findings highlight the significance of chromosomal analysis in the evaluation of infertility. The identification of structural rearrangements emphasizes the significance of examining both couples in infertility situations to identify potential genetic causes.

In patients presenting with recurrent pregnancy loss, the overall chromosomal anomaly rate was 2.7%. The prevalence of chromosomal abnormalities in this indication aligns with previous reports.^9^, ^48^, ^49^ Balanced translocations were the most frequent chromosomal abnormality observed. This finding is consistent with previous reports indicating that balanced translocations are associated with an increased risk of recurrent pregnancy loss.^9^, ^50^ Cytogenetic analysis is important and necessary for genetic counseling, implementation of appropriate reproductive strategies and taking precautions against unbalanced inheritance in their offspring. Polymorphism was detected in 213 patients with indication of recurrent pregnancy loss, and the most common of these was inv(9) polymorphism. In a clinical study on polymorphisms in this patient group, an increased frequency of polymorphic variants was reported compared to the control group.^51^ In a study, it was evaluated that the 46,XX,9q12h+ karyotype was responsible for the miscarriages in two cases that were clinically normal but had recurrent miscarriages.^52^ The comparison of chromosomal abnormalities in males and females within particular indications is a noteworthy aspect of our study. The findings showed significant disparities between males and females in the presence of chromosomal abnormalities in indications such as Down syndrome, infertility, and recurrent pregnancy loss.

In our study, the confirmed anomaly rate was 49.4% in females and 68.4% in males, and this difference was statistically significantly higher in males than in females. A meta‐analysis aligns with this study indicates that 1.3 times more males than females with Down syndrome, due to theories such as co‐orientation of non‐homologous in male meiosis, greater access of Y‐bearing sperm to the disomic ova for chromosome 21, or promotion of non‐disjunction by Y‐bearing sperm in the ova, and intrauterine selection against females.^53^, ^54^ In our study, male patients (6.7%) had statistically significantly higher rates of chromosomal abnormalities than female patients (1.5%). A recent study reported that chromosomal abnormalities in infertile patients were 8.19% in males and 7.82% in females.^41^ Our study showed that females (1.9%) have chromosomal abnormalities more often than males (1.3%) in patients with recurrent pregnancy loss. Most of the studies revealed that chromosomal abnormalities were predominant in females, which is consistent with our study.^49^, ^55^, ^56^ This may be because of the differences in the way male and female gametes develop and undergo cell division. Slow gamete development in females compared to males may increase chromosome defects in female gametes.^57^ These results point to potential differences between males and females in the genetic etiology and susceptibility to chromosomal abnormalities in specific clinical conditions. To completely comprehend the underlying mechanisms and implications of these gender differences, it is important to consider that further investigation is required.

This study has some limitations, even though it offers valuable data regarding the frequency and distribution of chromosomal abnormalities. The retrospective design and the reliance on data from a single medical genetics department may introduce selection bias and limit the generalizability of the findings. In addition, the study only considered the results of chromosomal analysis and did not take into account family segregation and other genetic testing methods, such as molecular cytogenetic or molecular approaches, which could have provided additional diagnostic information. Also, the available medical records were not suitable for us to access more information about patients and their families. Therefore, it was not possible to conduct a more detailed analysis.

To conclude, this retrospective study offers valuable insights into the frequency and distribution of chromosomal abnormalities in a diverse patient population. The results highlight the significance of chromosomal analysis in a range of clinical indications, such as recurrent pregnancy loss, intellectual disability, infertility, and particular syndromes. Comprehensive karyotyping is crucial for precise diagnosis and proper management, as it allows for the identification of numerical and structural chromosomal abnormalities, highlighting their importance. Genetic counseling, which includes family segregation in patients with detected chromosomal abnormalities, the risk of inheritance to subsequent generations, and the precautions to be taken against this risk, such as prenatal diagnosis, preimplantation genetic testing, etc., will also provide social benefit. Furthermore, examining chromosomal abnormalities in specific indications and comparing them between males and females underscore potential gender‐related variances that require additional research. Overall, this study, examining a large cohort, contributes to the existing literature and provides a basis for future research on chromosomal abnormalities and their implications in various indications.

## AUTHOR CONTRIBUTIONS

I.O.S. and H.A., both contributing equally, wrote and prepared the manuscript, S.A.P. wrote the manuscript, B.T., E.Y., M.D., M.Y., B.O.C., O.G.E., M.E., T.Y.K., and G.G. collect the data, A.K., H.P., M.T.O., N.B., Y.O., and M.D. reviewed the manuscript.

## CONFLICT OF INTEREST STATEMENT

The authors declare no conflicts of interest.

## Supporting information


Tables S1–S8

## Data Availability

The data that support the findings of this study are available from the corresponding author upon reasonable request.
