# Supplementary material for: Evaluation of chromosomal abnormalities in the postnatal cohort: A single‐center study on 14,242 patients
Source: J Clin Lab Anal. 2023 Dec 19;38(1-2):e24997. doi: 10.1002/jcla.24997 (PMC10829689; doi:10.1002/jcla.24997)
Supplement: Supplementary file 1 — Tables S1–S8 [file JCLA-38-e24997-s001.docx]

**Supplementary Tables**

**Table 1.** Karyotype Results in Patients with Amenorrhea

|  | Frequency (n) | Percent (%) |
| --- | --- | --- |
| 45,X | 1 | 1.7 |
| 45,X/46,X,del(p11.2-pter) | 1 | 1.7 |
| 45,X/46,X,i(X)(q10) | 1 | 1.7 |
| 45,X/46,XY | 1 | 1.7 |
| 46,X,del(X)(p22.1) | 1 | 1.7 |
| 47,XXX | 1 | 1.7 |
| 46,XX,del(X)(q13) | 1 | 1.7 |
| 46,XY | 1 | 1.7 |
| 46,XX | 52 | 86.7 |
| Total | n = 60 | 100.0% |

**Table 2.** Karyotype Results in Patients with Sex Development Disorders

|  | Frequency (n) | Percent (%) |
| --- | --- | --- |
| 45,X/46,XX/47,XXX | 1 | 1.3 |
| 45,X/47,XYY | 1 | 1.3 |
| 46,X,del(X)(p11.2) | 1 | 1.3 |
| 46,XX/47,XX+21 | 1 | 1.3 |
| 46,XX-Male | 1 | 1.3 |
| 46,XY-Female | 10 | 12.6 |
| 46,XX | 24 | 30.3 |
| 46,XY | 40 | 50.6 |
| Total | n = 79 | 100.0% |

**Table 3.** Karyotype Results in Patients with Turner syndrome

|  | Frequency (n) | Percent (%) |
| --- | --- | --- |
| 45,X | 30 | 9.8 |
| 45,X/46,XX | 6 | 2.0 |
| 45,X/46,XX/47,XXX | 3 | 1.0 |
| 45,X/46,X+mar | 1 | 0.3 |
| 46,X,del(X) | 3 | 0.9 |
| 46,X,i(X)(q10) | 4 | 1.3 |
| 46,X,i(X)(q10)/45,X | 5 | 1.6 |
| 46,XY | 1 | 0.3 |
| 46,XX | 254 | 82.7 |
| Total | n = 307 | 100.0% |

**Table 4.** Karyotype Results in Females and Males with Down Syndrome

| Female | Frequency (n) | Percent (%) | Male | Frequency (n) | Percent (%) | |
| --- | --- | --- | --- | --- | --- | --- |
| 46,XX | 121 | 25.9 | **46,XY** | 71 | 15.2 |  |
| 47,XX,+21 | 113 | 61.7 | **47,XY,+21** | 149 | 31.9 |  |
| 46,XX,t(14;21) | 1 | 0.2 | **46,XY,t(14;21)** | 1 | 0.2 |  |
| 46,XX,t(21;21) | 3 | 0.7 | **46,XY,t(21;21)** | 3 | 0.7 |  |
| 46,XX,+21,rob(13;22) | 1 | 0.2 | **46,XY,+21,t(13;14)** | 1 | 0.2 |  |
| 47,XX,+mar | 2 | 0.4 |  |  |  |  |
| 47,XXX | 1 | 0.2 |  |  |  |  |
|  | n = 242 | 51.8 % |  | n = 225 | 48.2% |  |

**Table 5.** Karyotype Results in Klinefelter syndrome

|  | Frequency (n) | Percent (%) |
| --- | --- | --- |
| 46,XY | 38 | 61.3 |
| 47,XXY | 23 | 37.1 |
| 47,XYY | 1 | 1.6 |
|  | n = 62 | 100.0 % |

**Table 6**. Karyotype Results in Females and Males with Infertility

| Female | Frequency (n) | Male | Frequency (n) |
| --- | --- | --- | --- |
| 45,X | 1 | **45,X/46,XY** | 4 |
| 45,X/46,XX | 5 | **45,XY,t(13;14)** | 5 |
| 45,X/46,XX/47,XXX | 1 | **45,XY,t(13;15)** | 1 |
| 46,XX,del(X)(q25) | 1 | **45,XY,t(14;21)** | 1 |
| 46,XX,t(12;20)(q20;q13.3) | 1 | **45,XY,t(15;21)** | 1 |
| 46,XX,t(5;6)(p15.2;p21.1) | 1 | **46,XY,del(16)** | 1 |
| 46,XX,t(6;16)(q27;q13) | 1 | **46,XY,del(Y)(q12)** | 1 |
| 47,XXX | 1 | **46,XY,del(22)(p11.2)** | 1 |
| 47,XXX/46,XX | 3 | **46,XY,inv(1)(q25;p36.3)** | 1 |
| 46,XX | 996 | **46,XY,inv(12)(p13.3;q22)** | 1 |
|  |  | **46,XY,t(1;10)(q25;q26.3)** | 1 |
|  |  | **46,XY,t(11;22)(q25;q13.1)** | 1 |
|  |  | **46,XY,t(15;17)(q23;q25)** | 1 |
|  |  | **46,XY,t(2;11)(q21.3;p15.2)** | 1 |
|  |  | **46,XY,t(5;11;13)(q15;p15.3;q34)** | 1 |
|  |  | **46,XY,t(5;19)(p11;p13.3)** | 1 |
|  |  | **46,XY,t(6;15)(p24;q21.2)** | 1 |
|  |  | **46,XY,t(8;13)(q22.3;q14.3)** | 1 |
|  |  | **47,XY,+mar** | 1 |
|  |  | **47,XXY** | 68 |
|  |  | **47,XYY** | 6 |
|  |  | **46,XY** | 1398 |
|  | n = 1011 |  | n = 1498 |

**Table 7.** Karyotype Results in Females and Males with Recurrent Pregnancy Loss

| Female | Frequency (n) | Male | Frequency (n) |
| --- | --- | --- | --- |
| 45,XX,rob(13;15)(p13;p13) | 1 | **45,XY,rob(13;15)(p13;p13),inv(Y)(p11.3;q11.22)** | 1 |
| 45,XX,rob(13;14)(p13;q13) | 6 | **45,XY,rob(13;14)(p11.2;p11.2)** | 1 |
| 45,XX,rob(13;22)(p11.2;p11.2) | 1 | **45,XY,rob(13;14)(p13;p13)** | 2 |
| 45,XX,rob(13;22)(p13;p13) | 1 | **45,XY,rob(13;22)(p13;p13)** | 1 |
| 45,XX,rob(14;15)(p13;p13) | 1 | **46,XY,t(4;10)(q34;q12.2)** | 1 |
| 45,XX,rob(14;15)(q10;q10) | 1 | **46,XY,t(4;5)(q35;q13)** | 1 |
| 45,XX,rob(14;21)(p13;p13) | 1 | **46,XY,t(5;11;13)(q15;p15.3;q34)** | 1 |
| 46,XX,t(10;19)(q23.2;q13) | 2 | **46,XY,t(5;11)(q35.2;q13,5)** | 1 |
| 46,XX,t(11;17)(p13;p13) | 1 | **46,XY,t(5;7) (p11.2;q35.2)** | 1 |
| 46,XX,t(15;17)(q23;q25) | 1 | **46,XY,t(7;9)(q31.1;p23)** | 1 |
| 46,XX,t(18;20)(p11.32;q12.1) | 1 | **46,XY,t(8;14)(p23;q24.3)** | 1 |
| 46,XX,t(2;17)(p36;p25) | 1 | **46,XY,t(Y;15)(q12;p13)** | 1 |
| 46,XX,t(2;4)(q37.3;q32.2) | 1 | **46,XY,del(13)(p11.1)** | 1 |
| 46,XX,t(2;13)(q21.1;q34),del(18)(12.2) | 1 | **46,XY,inv(7)(p12;q32)** | 1 |
| 46,XX,t(22;11)(q13.1;q25) | 1 | **46,XY/45,X/47,XXY** | 1 |
| 46,XX,t(3;7)(q29;q21.1) | 1 | **47,XXY** | 1 |
| 46,XX,t(4;9)(p14;q22.3) | 1 | **47,XYY** | 1 |
| 46,XX,t(5;10)(q33;q24) | 1 | **46,XY** | 2759 |
| 46,XX,t(5;18)(q13;q23) | 1 |  |  |
| 46,XX,t(6;11)(p25;q13) | 1 |  |  |
| 46,XX,t(6;17)(q23.2;p13) | 1 |  |  |
| 46,XX,t(7;14)(q11.2;p11.2) | 1 |  |  |
| 46,XX ,t(5;22)(p13.1;p12) | 1 |  |  |
| 46,XX, t(2;16)(p23;q23),21ps+ | 1 |  |  |
| 46,XX,del(13)(p11) | 1 |  |  |
| 46,XX,del(14)(q32.3) | 1 |  |  |
| 46,XX,del(22)(p12) | 3 |  |  |
| 46,XX,del(5)(p15.1) | 1 |  |  |
| 46,XX,inv(12)(p12;q3) | 1 |  |  |
| 46,XX,inv(12)(p13.3;q22) | 1 |  |  |
| 46,XX,inv(8)(p21.3;q11.21) | 1 |  |  |
| 45,X/46,XX | 11 |  |  |
| 45,X/46,XX/47,XXX | 3 |  |  |
| 47,XXX | 2 |  |  |
| 47,XXX,inv(9)(p11;q12) | 1 |  |  |
| 47,XXX/46,XX | 2 |  |  |
| 46,XX | 2975 |  |  |
|  | n = 3033 |  | n = 2777 |

**Table 8.** Polymorphism in Females and Males with Recurrent pregnancy loss

| Female | Frequency (n) | Male | Frequency (n) |
| --- | --- | --- | --- |
| 46,XX,13ps+ | 4 | **46,XY,13ps+** | 1 |
| 46,XX,14ps+ | 3 | **46,XY,14ps+** | 1 |
| 46,XX,15ps+ | 6 | **46,XY,15ps+** | 9 |
| 46,XX,16qh+ | 20 | **46,XY,16qh+** | 16 |
| 46,XX,1qh+ | 4 | **46,XY,1qh+** | 1 |
| 46,XX,21ps+ | 3 | **46,XY,21ps+** | 6 |
| 46,XX,22ps+ | 7 | **46,XY,22ps+** | 3 |
| 46,XX,6ph+ | 2 | **46,XY,9qh+** | 17 |
| 46,XX,9ph+ | 1 | **46,XY,inv(9)** | 34 |
| 46,XX,9qh+ | 20 | **46,XY,inv(Y)** | 2 |
| 47,XXX,inv(9) | 1 | **45,XY,rob(13;15)(p13;p13),inv(Y)(p11.3 ;q11.22)** | 1 |
| 46,XX,inv(9) | 51 |  |  |
|  | n = 122 |  | n = 91 |
